# Primary intraoral epithelioid hemangioendothelioma of the tongue: a case report and review of literature^⋆^

**DOI:** 10.1016/j.bjorl.2021.04.010

**Published:** 2021-05-13

**Authors:** Ekanayake Mudiyanselage Kanchana Medhavi Kumari Weerakoon, Kapu Gamage Kanchana Dewinda Kapugama, Bogahawatte Samarakoon Mudiyanselage Samadarani Siriwardena

**Affiliations:** a University of Peradeniya, Faculty of Dental Sciences, Department of Oral Pathology, Kandy, Sri Lanka; b University of Peradeniya, Faculty of Dental Sciences, Department of Oral and Maxillofacial Surgery, Kandy, Sri Lanka

## Introduction

Epithelioid hemangioendothelioma (EHE) is an uncommon borderline vascular tumor characterized by endothelial cells which has epithelioid morphology, first described by Weiss and Enzinger.^1^ The World Health Organization (WHO 2002) classification considered EHE as a locally aggressive tumor with metastatic potential.^2^

EHE arises most frequently in extremities,^1^ in relation to superficial and deep soft tissues. But also, cases have been reported in the lung, liver, bone, skin, head, and neck areas. EHE rarely occurs in the oral cavity.^1^, ^3^ The gingiva is the predominant site of occurrence.^2^ It usually can be seen in young and middle-aged males^4^ but there is a wide age distribution throughout adulthood.

Etiology and pathogenesis of EHE is still not clarified. Shabnum et al. stated that it is caused by a fusion gene, either a t(1;3)(p36;q23–5) reciprocal translocation between WWTR1 and CAMTA1 or a t(11;X)(q13;p11) between YAP1 TFE3.^4^

Intraoral EHE commonly presents as a painless solitary mass^4^, ^5^ which appears erythematous, purple pink to yellowish in color^3^ which had been present for a variable period of time. In some cases, it can be painful.^5^

Histologically, EHE is characterized by epithelioid or histiocytoid endothelial cells that proliferate forming small nests, short cords or solid nests embedded in a fibromyxoid matrix resembling hyaline cartilage.^1^ Epithelioid endothelial cells are polygonal or slightly spindled cells with eosinophilic cytoplasm and show cytoplasmic vacuolization.^1^ Factor VIII associated protein can be identified using immunohistochemical studies indicating a vascular origin.^1^, ^6^ Thus, clinicopathological and immunohistochemical characteristics are important in diagnosing EHE.

A wide local excision with adequate margins and close clinical followup has been reported as the treatment of choice for intraoral EHE because of the probability of local recurrrence.^3^, ^4^, ^6^ The behavior and outcome of EHE is unpredictable due to lack of criteria for diagnosis, differences in biological behavior according to site and age of occurrence.^5^ Local recurrence rate and metastatic rate are approximately 10% and 20% respectively.^1^ This report presents a case of EHE manifested in the tongue together with its histopathological and immunohistochemical studies.

## Case report

**Figure 1:**
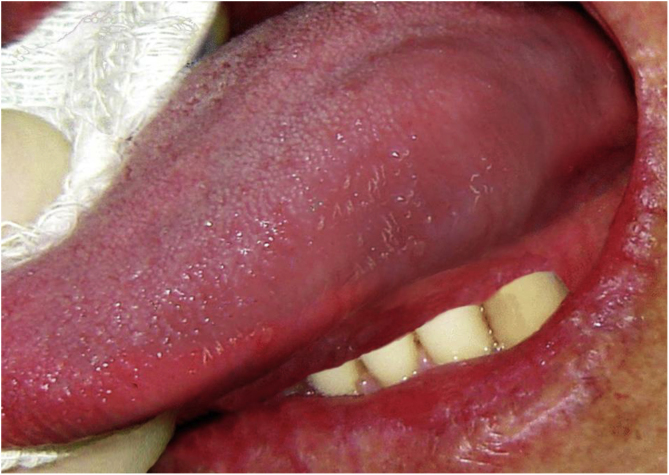
Clinical lesion presented as a swelling of lateral border of the tongue.

**Figure 2:**
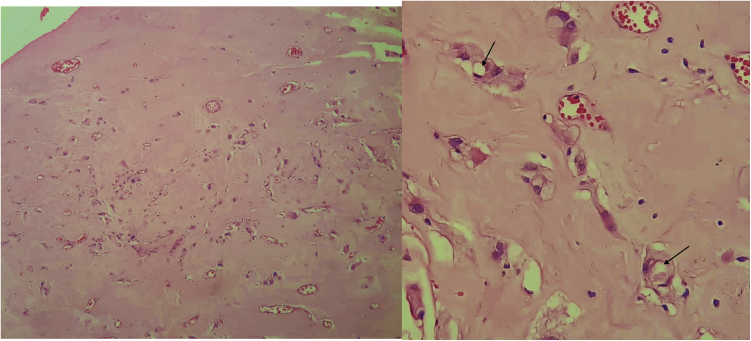
(A) Epithelioid cells dispersed within a densely collagenous stroma. Small capillaries are also present. (B) Epithelioid cells some of which show lumina formation (arrows).

**Figure 3:**
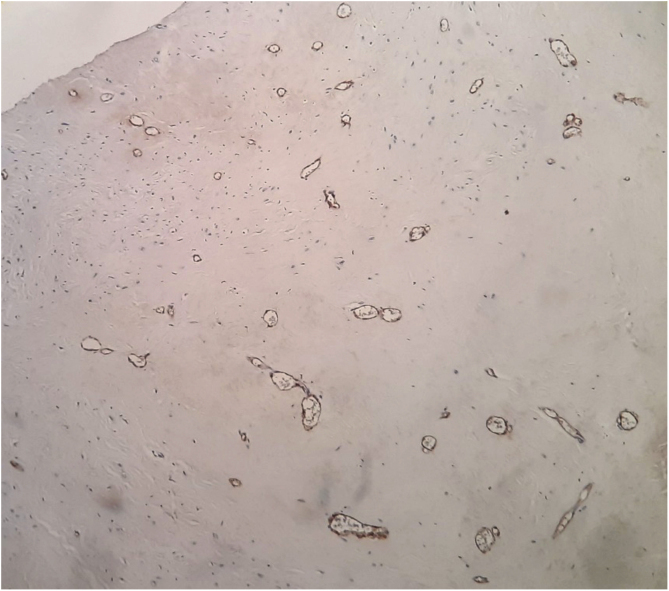
Factor VIII, immunohistochemical stain highlighting the vascular nature.

A 23-year-old male presented with a slowly growing painless lump on left side lateral border of the tongue for one year duration that had gradually increased in size. The patient had no relevant medical history and there was no history of alcohol consumption, betel chewing, smoking or trauma in the area. Also, the patient denied any history of similar kind of lesions. There were no significant extraoral findings. Intraoral examination revealed a well demarcated single, round, mobile nodule with yellowish erythematous appearance, in the lateral border of the tongue, measuring 1 × 1 cm (Fig. 1). The nodule was nontender and was soft to firm in consistency on palpation. It did not show ulceration, induration, or blanching effect. As it was benign in nature and a small lesion, an excisional biopsy was performed. Macroscopically it was 1.2 × 1.0 × 0.9 cm and histologically composed with small nets and cords of endothelial cells in a hyalinized stroma (Fig. 2A and B). Lumina formation was noted and it had a mild degree of atypia. Immunohistochemical stains for factor VIII confirmed the vascular origin and was diagnosed as epithelioid hemangioendothelioma (Fig. 3). The patient has been followed up for 26-months without local recurrences or metastasis although one margin was very closely excised.

## Discussion

The term hemangioendothelioma (HE) with the concept of vascular neoplasms has an intermediate or low-grade malignant potential and was first described by Borrmann.^2^ EHE is one of the three histological types (Kaposiform, Hobnail/Dabska-retiform, Epitheliod) of HE.^7^

The mean age of occurrence of EHE is 28-years with the age ranges from 4–76 years.^3^ According to Chi et al., EHE shows female predilection (M:F = 1:2.5)^3^ while a few other reports have reported male predilection.^4^ The age of the present patient also agrees with the majority of the previous literature. Intraoral sites of occurrence include gingival, palate, tongue, lip, buccal mucosa. Sreenivasan et al. stated that the predominant site of occurrence is gingiva.^7^ Common presentation of EHE is a painless solitary mass^4^ appearing erythematous, purple-pink to yellowish in color.^3^ It can clinically mimic, depending on the site, benign entities like cystic lesions (epidermoid/dermoid, deep seated mococeles, foregut cysts), benign tumors (fibromas, leiomyoma, rhabdomyoma, neurofibroma) or vascular tumors (pyogenic granuloma, EHE).^8^ In the literature most cases presented as a painless mass/nodule; incisional biopsy after other imaging modalities is necessary for proper diagnosis and staging. Investigations such as ultrasonography, magnetic resonance imaging, and computer assisted tomography could be done as well as isotopic scanning techniques. For centers that lack proper surveillance imaging, an incisional biopsy before definitive treatment is recommended. If the lesion is less than 1 cm, it is better to have an excision of the soft tissue lesion; however, depending upon the diagnosis, further definitive wide local excision is mandatory. Furthermore, regular close followup is recommended.

Oral cavity EHEs are isolated, and the best management is wide local excision. Some suggest a sentinel node biopsy as adjunct management.^8^ The case reported here was also clinically diagnosed as a benign lesion. Therefore, it is essential to have microscopic examination of such lesions to confirm the correct diagnosis.

Histologically the presence of small nests and cords of endothelial cells, cytoplasmic vacuolization, fibrotic and hyalinized stroma are some of the features that are shared by the present case and previous cases.^1^, ^3^, ^5^ These epithelioid endothelial cells resemble many of the features of normal endothelium, including the presence of factor VIII protein, numerous junctional attachments, pinocytotic vesicles, investing basal lamina and Weibel-Palade bodies, but differ by their round nuclei and superabundant cytoplasm crowded with cytofilaments.^1^ Features such as cytological atypia, cellular pleomorphism, high mitotic activity (>1/10 high power fields), increased number of spindling cells than epithelioid cells, focal necrosis, metaplastic bone formation within the tumor indicate potential for malignant behavior.^1^ The case reported here showed a mild degree of cellular atypia but none of other features mentioned above, which is suggestive of a less aggressive variant of the tumor. Although the above features have been described, no consistent clinical or histological criteria for predicting the biological behavior of malignant EHE has been identified. Microscopic differential diagnosis includes metastatic carcinoma, angiosarcoma and hemangiopericytoma.^3^ Presence of less pleomorphism, less mitotic activity, and less cytological atypia in EHE aids differentiation from angiosarcoma^9^ and metastatic carcinoma.^1^

In addition to histopathology, immunohistochemical studies for markers such as CD31, CD34, factor VIII related antigen,^3^, ^5^, ^7^ VEGF,^6^ PCNA,^6^ help to differentiate EHE from other entities. Factor VIII is highly variable in its distribution but correlation of the degree of differentiation with the presence of factor VIII is not clearly identified. A recent study done by Naqvi et al. demonstrated the use of podoplanin in differentiating EHE from non-vascular tumors.^8^

Treatment of choice has been suggested as wide local excision with adequate margins due to probability of recurrence.^3^, ^4^, ^6^ It is difficult to make fixed therapeutic recommendations because of its borderline histopathologic nature.^1^ Lesions having high malignant potential should be treated by radical surgery while benign lesions should be completely excised with a small margin of normal tissue.^1^ Inadequate or incomplete excision can lead to local recurrence.^9^ In the present case, the tumor is closely excised which opens the possibility of local recurrence. The effect of adjuvant chemotherapy and radiotherapy is still not confirmed. Close long term follow-up is recommended as recurrences after a long disease-free period has been reported.^3^, ^9^

Weiss and Enzinger observed the local recurrence rate of 10% and metastatic rate of 20% in their study.^1^ In the oral cavity EHEs, local recurrences have been reported but no local or distant metastasis when compared with other sites EHEs, suggesting a less aggressive nature of intra-oral EHE.^4^ Followup clinical examination should include evaluation of regional lymph nodes. Previously reported cases also showed no mortality in head and neck EHE. Table 1 summarizes EHE of the tongue that were documented in the literature. Depending of the site, EHE shows different behavior, indicating that the location is best indicator of potential treatment options. Furthermore, local excision with close followup may be the appropriate management for EHE of the tongue.

**Table 1:** Summary of EHE in the literature.

Author	Age/sex	Site	Clinical presentation	Follow-up	Year of publication
Marrogi et al.^5^	36F	Right side lateral border of the tongue	2-months, painful, 0.2 cm	17-months without recurrence	1991
Molina Palma et al.^4^	65F	Tongue	2-months, 0.5 cm	21-months without recurrence	2002
Uehara et al.^6^	72M	Dorsal tongue	2-months duration, painless, 0.7 × 0.7 cm, moderately firm	No information	2006
Naqvi et al.^8^	71M	Tongue	1cm	No information	
Sun et al.^3^	17M	Tongue	2-months, soft in consistency, 0.5 cm	19-months without recurrence	2007
Sun et al.^3^	21M	Tongue	2-months, reddish swelling, 0.5 cm	2-years without recurrence	2007
Sun et al.^3^	34M	Tongue	4-months, 1 cm	6-years without recurrence	2007
Sun et al.^3^	46M	Tongue	1.2 cm	Recurrence after 4-months	2007
A Sawair ^10^	1M	Tongue	6-months, 1 × 0.9 cm	12-years alive, no recurrence	2008
A Sawair ^10^	3F	Tongue	2-months, multiple nodules, 2 cm in length	4-years alive, no recurrence	2008
Present case	23M	Left side lateral border of the tongue	1-year, painless, reddish/yellowish lump, 1 × 1cm	26-months without recurrence	

## Conclusion

EHE is an intermediate grade vascular neoplasm that requires critical therapy and followup. Uncertainty in determining malignant risk indicates the need for a standard clinical and histological criterion to precisely predict the behavior of malignant EHE. Due to the borderline nature, overlapping morphological features with certain other disorders and rarity, it is often misdiagnosed clinically. Hence, familiarity with its characteristic clinical and pathological features will lead to accurate diagnosis, appropriate management, and consequently reveal a higher incidence of this tumor.

## Patient perspective

The patient was informed about the treatment options and possible postoperative complications. Informed consent was obtained for both surgical procedure and for research.

## Data availability

All data were included in the manuscript.

## Conflicts of interest

The authors declare no conflicts of interest.
